# A Novel CpG Methylation Risk Indicator for Predicting Prognosis in Bladder Cancer

**DOI:** 10.3389/fcell.2021.642650

**Published:** 2021-09-01

**Authors:** Yufeng Guo, Jianjian Yin, Yuanheng Dai, Yudong Guan, Pinjin Chen, Yongqiang Chen, Chenzheng Huang, Yong-Jie Lu, Lirong Zhang, Dongkui Song

**Affiliations:** ^1^Department of Urology, The First Affiliated Hospital & Academy of Medical Sciences, Zhengzhou University, Zhengzhou, China; ^2^Department of Pharmacology, School of Basic Medical Sciences, Zhengzhou University, Zhengzhou, China; ^3^Centre for Cancer Biomarkers and Biotherapeutics, Barts Cancer Institute, Queen Mary University of London, London, United Kingdom

**Keywords:** DNA methylation, prognosis, LASSO, SVM-RFE, bladder cancer, machine learning

## Abstract

**Purpose:**

Bladder cancer (BLCA) is one of the most common cancers worldwide. In a large proportion of BLCA patients, disease recurs and/or progress after resection, which remains a major clinical issue in BLCA management. Therefore, it is vital to identify prognostic biomarkers for treatment stratification. We investigated the efficiency of CpG methylation for the potential to be a prognostic biomarker for patients with BLCA.

**Patients and Methods:**

Overall, 357 BLCA patients from The Cancer Genome Atlas (TCGA) were randomly separated into the training and internal validation cohorts. Least absolute shrinkage and selector operation (LASSO) and support vector machine-recursive feature elimination (SVM-RFE) were used to select candidate CpGs and build the methylation risk score model, which was validated for its prognostic value in the validation cohort by Kaplan–Meier analysis. Hazard curves were generated to reveal the risk nodes throughout the follow-up. Gene Set Enrichment Analysis (GSEA) was used to reveal the potential biological pathways associated with the methylation model. Quantitative real-time polymerase chain reaction (PCR) and western blotting were performed to verify the expression level of the methylated genes.

**Results:**

After incorporating the CpGs obtained by the two algorithms, CpG methylation of eight genes corresponding to TNFAIP8L3, KRTDAP, APC, ZC3H3, COL9A2, SLCO4A1, POU3F3, and ADARB2 were prominent candidate predictors in establishing a methylation risk score for BLCA (MRSB), which was used to divide the patients into high- and low-risk progression groups (*p* < 0.001). The effectiveness of the MRSB was validated in the internal cohort (*p* < 0.001). In the MRSB high-risk group, the hazard curve exhibited an initial wide, high peak within 10 months after treatment, whereas some gentle peaks around 2 years were noted. Furthermore, a nomogram comprising MRSB, age, sex, and tumor clinical stage was developed to predict the individual progression risk, and it performed well. Survival analysis implicated the effectiveness of MRSB, which remains significant in all the subgroup analysis based on the clinical features. A functional analysis of MRSB and the corresponding genes revealed potential pathways affecting tumor progression. Validation of quantitative real-time PCR and western blotting revealed that TNFAIP8L3 was upregulated in the BLCA tissues.

**Conclusion:**

We developed the MRSB, an eight-gene-based methylation signature, which has great potential to be used to predict the post-surgery progression risk of BLCA.

## Introduction

Bladder cancer (BLCA) is one of the most common cancers. Seventy percent of cases present as non-muscle invasive lesions (NMIBCs), and approximately 25–75% of high-risk NMIBC patients progress to muscle invasive cancer (MIBC) and then to metastatic cancer (Siegel et al., 2020). Patients with MIBC have a poorer prognosis due to tumor recurrence and progression, and their 5-year survival rate is 25–60%. Biomarkers that can credibly evaluate the disease prognosis and patient survival would have tremendous benefits in guiding the individualized management of patients. Epigenetic modifications of DNA methylation can be identified by high-throughput analysis, and they regulate gene expression, which can contribute to the diagnosis, prevention, and treatment of diseases. Abnormal methylation generally occurs in early cancer and influences cancer progression (Ibrahim et al., 2011).

Because alterations in aberrant methylation are relatively stable and may be reversible therapeutically, considerable attention has been focused on them recently (Garcia-Manero et al., 2013). Tumor initiation and progression in BLCA patients is thought to be associated with abnormal DNA methylation (Kim and Kim, 2009; Besaratinia et al., 2013; Kandimalla et al., 2013). A previous study showed that NMIBC patients without prostate cancer susceptibility candidate (PRAC) methylation have a higher risk of recurrence or progression than those with methylation (Kim et al., 2015), and RUNX family transcription factor 3 (RUNX3) methylation was identified as a potential biomarker associated with overall survival (OS) by Jeong et al. (2011). However, there are still few methylation markers widely accepted for BLCA. The identification of reliable markers has become a feasible method with the emergence of high-throughput technology. CpG methylation as a biomarker predicting OS has been demonstrated by several previous genome-wide studies, but it does not predict progression-free survival (PFS) (Kawamoto et al., 2006; Luo et al., 2014; Shivakumar et al., 2017). Because the OS of NMIBC patients whose tumors are limited to the urothelial layer is favorable after treatment by transurethral resection, especially patients in G1/G2 stage, PFS more accurately reflects the biological behavior of BLCA (Beukers et al., 2015). Consequently, identifying the potential prognostic biomarker in predicting the risk of BLCA recurrence and/or after initial surgical treatment will be critical to maximally control cancer progression while avoiding overtreatment.

In this study, we successfully identified and validated progression-related CpGs in BLCA. Here, we analyzed DNA methylation data from 450K chips from The Cancer Genome Atlas (TCGA)-BLCA database by utilizing machine learning and built a predictive model from the methylation risk score for BLCA (MRSB) with eight specific CpGs for predicting the PFS of BLCA patients. We revealed the time node of adverse events after resection, thus allowing for more efficient treatment of patients to prevent a poor outcome of high-risk patients. We further demonstrated that the mRNA and protein levels of the MRSB component-related gene TNFAIP8L3 were prominently upregulated in BLCA tissues compared to adjacent tissues. In short, our study identified a prognostic panel, which provides novel insight into cancer progression and the opportunity of stratified therapeutic strategy for patients with BLCA.

## Materials and Methods

### Patients and Tumor Samples

Paired cancer and adjacent tissue samples from 18 patients were collected between July 2017 and June 2019 at the First Clinical Hospital of Zhengzhou University (ZZU cohort). None of the patients had previously received any special treatments. The project was approved by the Ethics Committee of Zhengzhou University, and all patients signed informed consent forms. Patient tissues were stored in liquid nitrogen until they were used for the detection of mRNA and protein expression levels.

### Public Data Collection and Grouping of Patients

**FIGURE 1:**
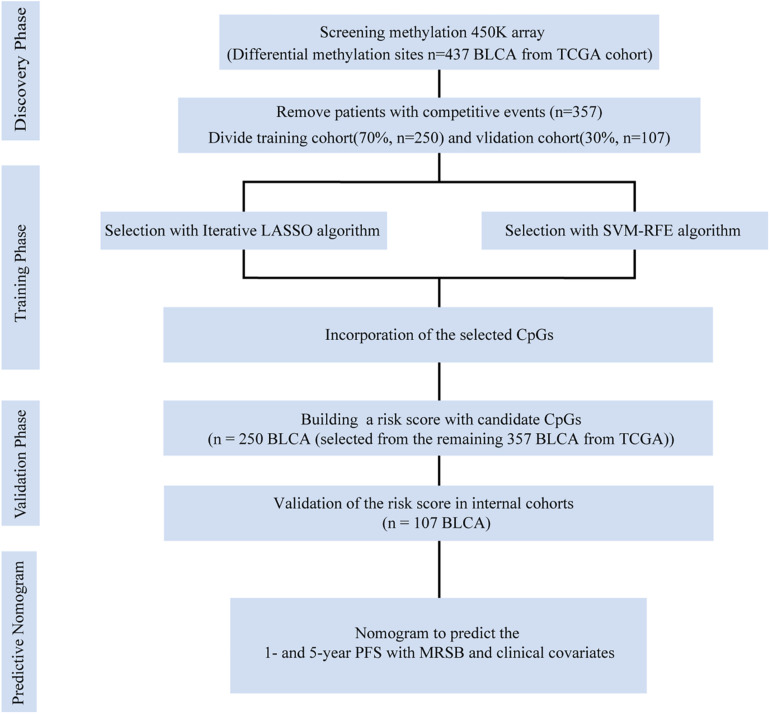
Data generation and analysis process of this study. The differentially presented CpGs between cancer and normal tissues in the TCGA bladder cancer cohort were firstly identified. After excluding competitive event patients, SVM-RFE and LASSO algorithms were used to identify candidate methylation sites and to incorporate the results. Multivariate Cox analysis was performed to establish the prognostic model: the MRSB was validated in the internal validation cohort. Finally, nomograms were established with MRSB and clinical covariates. TCGA, The Cancer Genome Atlas; SVM-RFE, support vector machine-recursive feature elimination; LASSO, least absolute shrinkage and selector operation; BLCA, bladder cancer; MRSB, methylation risk score for bladder cancer.

The research protocol is illustrated in Figure 1. BLCA patients’ raw DNA methylation data based on the Illumina Human Methylation 450 (450K) Bead Chip were obtained from TCGA.^1^ Among a total of 485,578 CpG sites with annotation, only 395,985 CpG sites corresponded to known genes. Thus, all 395,985 CpG sites were finally selected for our analysis. Based on the BLCA patients with complete clinical information, we designed the inclusion criteria for groups of patients. Patients who died of non-cancer-related events were excluded. Finally, 357 BLCA patients were included in our study. According to the methods used in previous research (Simon et al., 2003), the 357 BLCA patients from the TCGA were divided randomly into training and internal validation groups by a professional programmer utilizing a random allocation sequence to implement computerized random allocation.

### Screening of Methylated CpG Sites

The differentially expressed CpG sites between BLCA patients and adjacent normal tissue were selected using the “limma” package (Ritchie et al., 2015; Li et al., 2019) with significant cutoff values of the adjusted *p* < 0.01 and | log2-fold change (FC)| > 0.2 in R software (version 3.6.4), which is a more stringent standard than that used in previous studies, to determine the differential CpG sites (Ma et al., 2019). Through Cox regression screening, differentially expressed CpG sites were found to be related to prognosis (log-rank tests *p* < 0.05) (George et al., 2014) using the “survival” R package (Williams et al., 2017). CpG sites that conformed to the criteria described above were selected to train the model.

### Machine Learning for the Candidate CpGs

**FIGURE 2:**
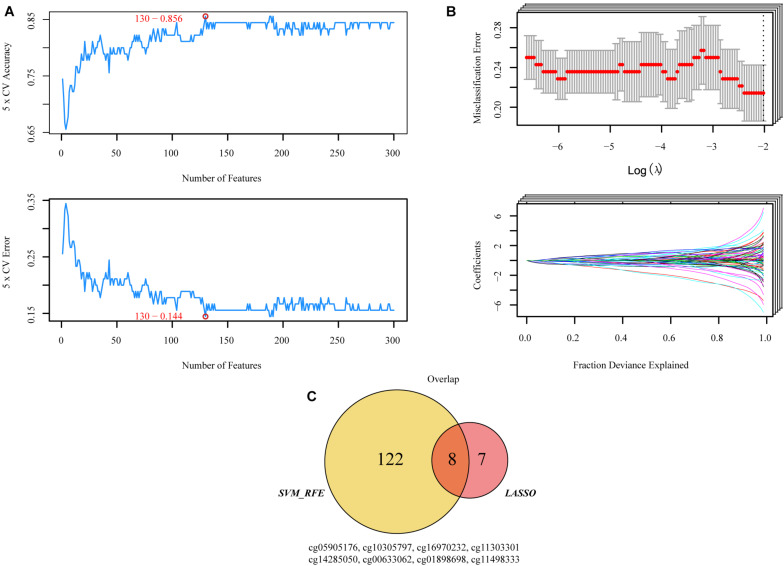
Two algorithms were used for selecting the candidate CpGs. **(A)** The number of features with the optimal accuracy and the lowest error rate under fivefold cross validation in SVM-RFE. **(B)** LASSO algorithms in the discovery cohort. **(C)** Incorporation of CpGs methylation that were selected from the LASSO and SVM-RFE algorithms in the discovery cohort. SVM-RFE, support vector machine-recursive feature elimination; LASSO, least absolute shrinkage and selector operation.

CpG sites conforming to the criteria described above were used to participate in machine learning. “Glmnet” R packages were utilized to implement the least absolute shrinkage and selector operation (LASSO) algorithm (Tibshirani, 2011), and “e1071” R packages were executed to support vector machine-recursive feature elimination (SVM-RFE) (Guo et al., 2014; Huang et al., 2014). The final candidate CpGs were obtained by the intersection of the results from the two algorithms (Figures 2A–C).

### Construction of the Methylation Risk Score

**FIGURE 3:**
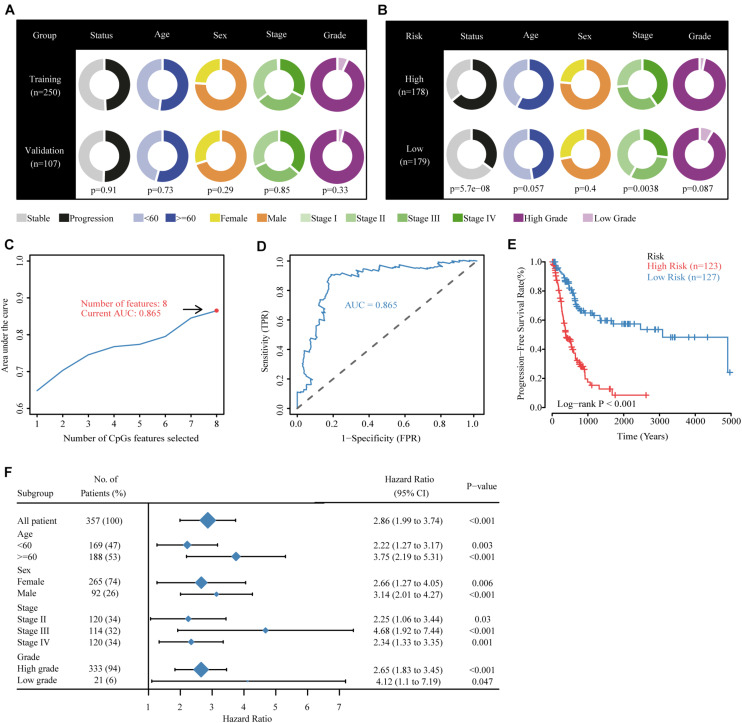
Building the methylation risk score for bladder cancer (MRSB) in the training cohort. **(A)** Clinical characteristics of the training and validation cohorts. **(B)** Clinical characteristics of the high- and low-risk groups identified by MRSB. **(C)** Eight CpG predictive features had the highest discriminative power for high-risk and low-risk patients. **(D)** Area under the curve of the receiver operating characteristic curve for MRSB to distinguish between high- and low-risk groups. **(E)** Progression-free survival was significantly different between the high- and low-risk groups. **(F)** The prognostic value of MRSB in patient subgroups with different patient and clinical features.

All possible stepwise increases in the amounts of candidate CpGs were tested from one to eight signatures to obtain the best classification accuracy of patients in the high- and low-risk groups (Figures 3A,B). Next, Cox proportional hazards modeling was conducted to construct a CpG predictive signature on candidate CpGs obtained from the previous test with the optimal number of signatures. Receiver operating characteristic analysis and multivariate Cox regression were performed with the “survivalROC” R package (Heagerty et al., 2000). The “Muhaz” package was used to calculate the kernel-smoothing hazard rate function for the risk component in PFS and OS (Müller and Wang, 1994; Li et al., 2020).

### Building a Predictive Nomogram

Decision curve analysis was executed to select the optimal clinical variables to incorporate for constructing a nomogram (Vickers and Elkin, 2006). The concordance index was used to validate the efficiency of the nomogram, and calibration plots were graphically explored. The “survival” and “rms” R packages were used.

### Real-Time Quantitative Polymerase Chain Reaction

TRIzol reagent (Invitrogen, United States) was utilized to extract total RNA from the tissues, and it was reverse transcribed following the manufacturer’s protocol (Takara Bio, Japan). Real-time quantitative polymerase chain reaction (RT-qPCR) assays were performed using the PowerUp SYBR-Green master mix kit (Thermo Fisher Scientific, United States) and the QuantStudio 6 System (Thermo Fisher Scientific, United States). The following primers were used: TNFAIP8L3 forward primer: ATTGATGACACCAGCAGCGA; reverse primer: GAGGAACTCCACATCGGCAA. Glyceraldehyde 3-phosphate dehydrogenase (GAPDH) forward primer: GACCTGACCTGCCGCCTA; reverse primer: AGGAGTGG GTGTCGCTGT. mRNA expression was normalized to GAPDH, and the data were analyzed using the comparative Ct method (2^−ΔΔ*Ct*^).

### Western Blotting

Total protein was extracted utilizing radioimmunoprecipitation assay (RIPA) buffer from the tissues of the ZZU BLCA patients. Following the extraction, bicinchoninic acid (BCA) assays (Beyotime, China) were performed to quantify all proteins. Equal amounts of protein samples were separated by 10% sodium dodecyl sulfate polyacrylamide gel electrophoresis (SDS-PAGE) and then transferred to polyvinylidene difluoride (PVDF) membranes (Millipore, United States). The membranes were blocked with 5% non-fat milk/TBST for 2 h. Then, the membranes were incubated with primary antibodies at 4°C overnight with the following antibodies: anti-TNFAIP8L3 (1:1,000; Invitrogen, United States) and anti-GAPDH (1:10,000; Proteintech, United States). After washing the membranes with Tris-buffered saline Tween-20 (TBST) three times, the membranes were further incubated with secondary antibodies [alkaline phosphatase-conjugated AffiniPure goat anti-rabbit IgG (H+L) (1:10,000; Proteintech, United States) or alkaline phosphatase-conjugated AffiniPure goat anti-mouse IgG (H+L) (1:10,000; Proteintech, United States)] for 2 h at 37°C. The immunoreactive bands were visualized using an enhanced chemiluminiscence (ECL) system (FluorChem E; ProteinSimple, United States).

### Pathway Enrichment Analysis for the MRSB Associated Genes

Gene Set Enrichment Analysis (GSEA) was executed to identify potential biological pathways/processes affected by the MRSB DNA methylation model and corresponding genes (Mootha et al., 2003; Subramanian et al., 2005). Ranking the samples by the expression of the methylated genes, samples with expression of the genes greater than 75% of all samples were defined as the high expression group, and those with expression <25% of all samples were defined as the low expression group. The gene sets of ‘‘kegg pathway’’ and ‘‘hallmarks of cancer’’ were acquired from ‘‘GSEA Molecular Signatures Database.’’^2^ Enrichment analysis was performed by “fgsea” and “clusterProfiler” R package (Yu et al., 2012).

### Statistical Analysis

All statistical analyses were executed in R software (Version 3.6.4). The “survival” R package was executed to perform the Kaplan–Meier survival analysis and log-rank test. PFS was measured as the time when patients lived with the disease during which it did not worsen until the last follow-up after treatment. OS was defined as the date of diagnosis or the start of treatment to death or the last follow-up. Recurrence-free survival (RFS) was defined as the time from treatment until disease recurrence, metastasis, or last follow-up. Statistical significance was defined as *p* < 0.05 unless specified otherwise.

## Results

### Patient Characteristics and Grouping

Following the protocol illustrated in Figure 1, samples containing competing events were removed, which ended up with 357 BLCA patients with median follow-up time of 18.2 months (range 0.43–168.3). At the final point of follow-up, 49.58% of the patients with BLCA (177 of 357) experienced disease progression, and 35.29% of the patients (126 of 357) died. For the overall cohort, the 1- and 5-year PFS rates were 74.32 and 53.70%, and the 1- and 5-year OS rates were 57.89 and 38.78%, respectively. Patients whose disease progressed within 1 year were defined as the high-risk group (*n* = 110), and those who had five or more years of follow-up and had no disease progression events were defined as the low-risk group (*n* = 30).

### Selection of Candidate CpGs

Both SVM-RFE and iterative LASSO were used to identify the most significant CpGs for classifying patients into high- and low-risk progression in the training group. A total of 130 CpGs (Supplementary Table 1) were identified by the SVM-RFE algorithm (Figure 2A). Using the iterative LASSO algorithm, 15 CpGs (Supplementary Table 2) that appeared more than 500 times in the 1,000 iterations (Figure 2B) were considered consensus CpGs that distinguished high- from low-risk groups. After incorporating the CpGs obtained by the two algorithms, eight CpGs corresponding to TNFAIP8L3, KRTDAP, APC, ZC3H3, COL9A2, SLCO4A1, POU3F3, and ADARB2 identified by both algorithms were selected as the final risk signatures (Figure 2C).

### Building the Methylation Risk Score

The TCGA were divided randomly into training (250 patients, 70%) and internal validation (107 patients, 30%) groups using a random allocation sequence, and there were no significant differences in progression risk status, age, sex, cancer stage, and grade between the training and validation cohorts (Figure 3A). To better evaluate the efficiency of candidate CpGs in predicting progression, we applied a multivariate Cox model to obtain the coefficients weighted for building a MRSB in the training sample cohort. The following formula was used to calculate the MRSB: risk score = (−0.919 × methylation level of TNFAIP8L3) + (−1.383 × methylation level of KRTDAP) + (−1.071 × methylation level of APC) + (−3.213 × methylation level of ZC3H3) + (3.348 × methylation level of COL9A2) + (−2.626 × methylation level of SLCO4A1) + (1.522 × methylation level of POU3F3) + (0.803 × methylation level of ADARB2). Using the median MRSB cutoff point of 1.038, 123 patients were assigned to the high-risk (>1.038) group, and 127 patients were assigned to the low-risk (>1.038) group (Figure 3B). With this cutoff threshold, the area under the receiver operating characteristic curve (AUC) to predict high progression risk patients from the training cohort was 0.864 (Figures 3C,D), and PFS was significantly different between the high- and low-risk patients (*p* < 0.001, Figure 3E). We further assessed the prognostic value of MRSB in patient subgroups with different patient and clinical features and found that a high MRSB significantly increased patient BLCA progression risk in all subgroups (Figure 3F).

### Validating the Methylation Risk Score

**FIGURE 4:**
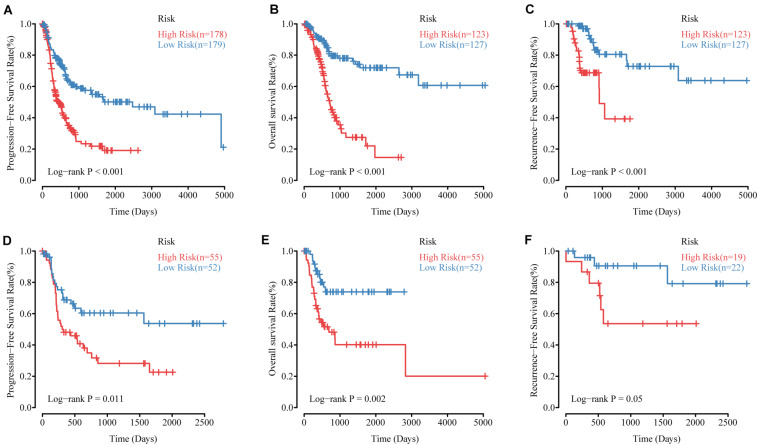
The performance of MRSB in predicting progression, survival and recurrence. **(A)** Progression-free survival in all patients. **(B)** Overall survival in the training cohort. **(C)** Recurrence-free survival in the training cohort. **(D)** Progression-free survival in the validation cohort. **(E)** Overall survival in the validation cohort. **(F)** Recurrence-free survival in the validation cohort. MRSB, methylation risk score for bladder cancer.

To validate the effectiveness of the MRSB, we performed validation analysis in the internal validation cohort (*n* = 107). MRSB classified the internal validation cohort into high-risk (*n* = 55, 51.4%) and low-risk (*n* = 52, 48.6%) groups with significant differences in PFS (log-rank *p* = 0.011, Figure 4D). Meanwhile, the same results were seen in all of the patients (high-risk *n* = 178, 49.1%, low-risk *n* = 179, 50.1%, log-rank *p* < 0.001, Figure 4A). Furthermore, we found that the predictive signature maintained its discriminative efficacy for OS and RFS of the patients in the training cohort (Figure 4B, OS: *p* < 0.001 and Figure 4C, RFS: *p* < 0.001) and the internal validation cohort (Figure 4E, OS: *p* = 0.002 and Figure 4F, RFS: *p* = 0.05).

### Kernel-Smoothing Hazard Rate Function

**FIGURE 5:**
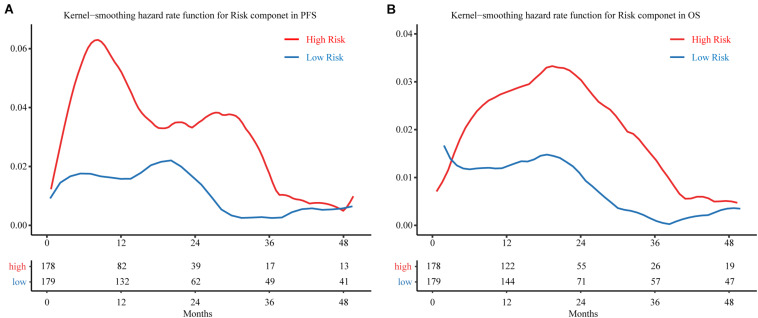
Hazard curves revealing the time of cancer progression. Smoothed hazard estimates for the presence of a risk component in **(A)** PFS and **(B)** OS. The red line represents patients in the high-risk group, and the blue line represents patients in the low-risk group. The table below the curve gives the number of patients without observed endpoint event at different follow-up cut-of time. PFS, progression-free survival; OS, overall survival.

A kernel-smoothing hazard rate function was used to reveal the time to cancer progression. The risk increased steeply toward the first peak at approximately 9–10 months after treatment for the MRSB high-risk group, and the second peak occurred at approximately 30 months after resection; however, there was no noteworthy peak for the MRSB low-risk group during the follow-up period (Figure 5A). For the OS of the patients, a prominent peak approximately 20 months after resection was significant, which was slightly later than the peak for the time of cancer progression in the MRSB high-risk group (Figure 5B).

### Building a Predictive Nomogram

**FIGURE 6:**
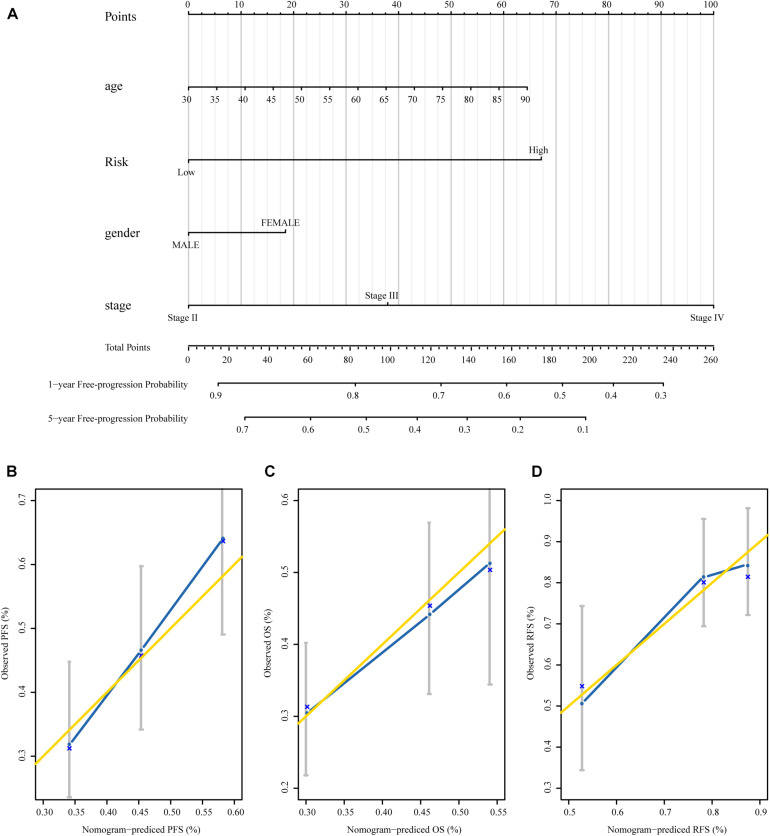
The nomograms based on MRSB are reliable predictors for the prognosis of BLCA patients. **(A)** Nomogram to predict the 1- and 5-year PFS. **(B)** Calibration curve for the PFS nomogram model in the TCGA BLCA cohort. **(C)** Calibration curve for the OS nomogram. **(D)** Calibration curve for the RFS nomogram model. The gold line represents the ideal nomogram, and the blue line represents the observed nomogram (for the OS and RFS nomogram see the Supplementary Material). MRSB, methylation risk score for bladder cancer; BLCA, Bladder cancer; PFS, progression-free survival; TCGA, The Cancer Genome Atlas; OS, overall survival; RFS, recurrence-free survival.

To establish a clinical valuable prognostic biomarker based on our MRSB to predict the individual risk of disease progression, we developed a predictive model by combining MRSB and common clinical covariates using a nomogram. Based on the decision curve, we found that pathologic tumor stage was a better evaluation factor than histological grade (Supplementary Figure 1). We created a nomogram with predictors including MRSB, pathologic tumor stage, age, and sex of the patients to predict the 1- and 5-year PFS (Figure 6A). Using the same approach, nomograms were also generated to predict the 1- and 5-year OS and RFS, respectively (Supplementary Figures 2, 3). The calibration graphs of the 5-year PFS rate, 5-year OS rate, and 3-year RFS rate performed well (Figures 6B–D).

### Confirmation of the Gene Expression Changes of MRSB DNA Methylation Model

**FIGURE 7:**
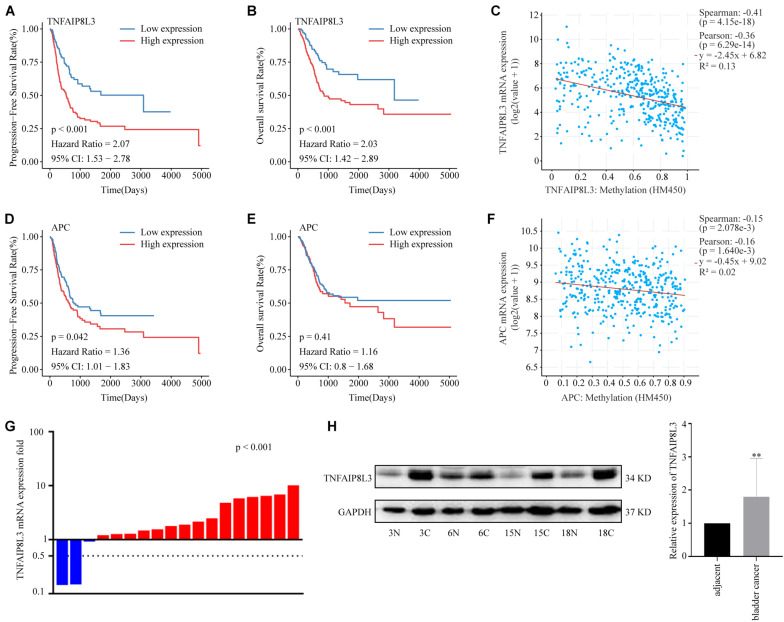
Expression patterns of MRSB component-related genes. **(A)** Progression-free survival curves according to the presence of the MRSB component-related gene TNFAIP8L3. **(B)** Overall survival based on TNFAIP8L3 expression. **(C)** Negative correlation between TNFAIP8L3 expression and the methylation level. **(D)** Progression-free survival and **(E)** overall survival curves based on the expression of APC. **(F)** Negative correlation between APC expression and the methylation level in the ZZU cohort. **(G)** The mRNA expression levels of TNFAIP8L3 in the cancer and adjacent tissues (*p* < 0.001, ZZU cohort). **(H)** The protein expression levels of TNFAIP8L3, **p < 0.001. MRSB, methylation risk score for bladder cancer; ZZU, Zhengzhou University.

Methylation risk score for BLCA (MRSB) component-related genes were defined as the genes at which the probe closest to the transcription start site (TSS) was located based on the university of California Santa Cruz (UCSC) genome browser known-gene list. To evaluate the potential contribution of the MRSB component-related genes in BLCA progression, we analyzed the RNA-seq data of those genes in BLCA samples in correlation with patient PFS and OS in the TCGA cohort (Supplementary Figure 4). We found that high levels of TNFAIP8L3 were significantly correlated with poor PFS/OS (*p* < 0.001) rates (Figures 7A,B) and high levels of APC were associated with poor PFS (*p* = 0.042), although not with OS (*p* = 0.41) (Figures 7D,E) in BLCA patients. Meanwhile, both expression levels of TNFAIP8L3 and APC were significantly negatively correlated with their methylation status (Figures 7C,F). We further experimentally confirmed using our own sample cohort (ZZU cohort) that the mRNA and protein expression levels of TNFAIP8L3 were significantly upregulated in BLCA tissues (Figures 7G,H).

### Biological Pathways/Processes Affected by the MRSB DNA Methylation Model

**FIGURE 8:**
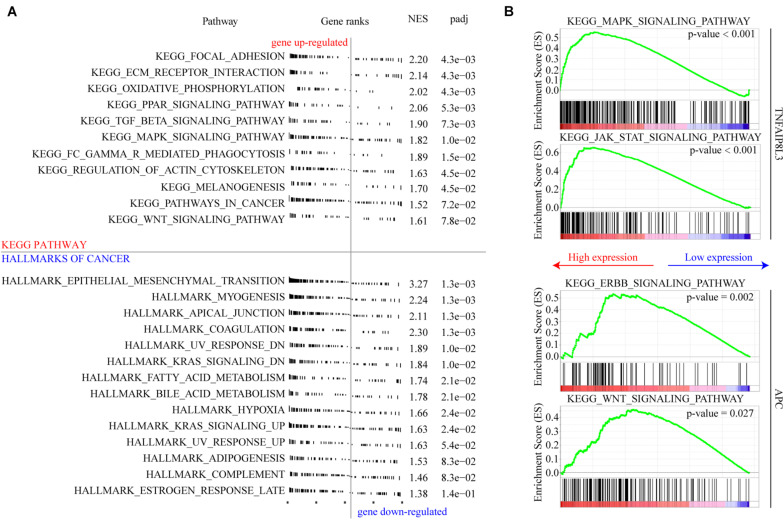
Cellular pathways/processes affected by MRSB methylation model. **(A)** Gene Set Enrichment Analysis showing the signature in the context of gene sets representative for Kyoto Encyclopedia of Genes and Genomes (KEGG) pathway and cellular process from hallmarks of cancer. **(B)** MAPK signaling pathway and JAK-STAT signaling pathway were significantly enriched in TNFAIP8L3 overexpression cases. ERBB signaling pathway and WNT signaling pathway were enriched in APC overexpression cases. MRSB, methylation risk score for bladder cancer.

We used GSEA to explore the biological effects mediated by the methylation model and the corresponding genes. We found that several gene sets associated with tumor progression were enriched, and the associated genes were upregulated in the high-risk group (Figure 8A). Focal adhesion, extracellular matrix (ECM) receptor interaction, and epithelial mesenchymal transition which are all associated with cancer invasion and metastasis, are the top three cellular processes significantly affected by the MRSB DNA methylation model. The genes of MAPK signaling pathway and JAK-STAT signaling pathway were significantly enriched when TNFAIP8L3 expression was upregulated. When APC expression was upregulated, ERBB signaling pathway and WNT signaling pathway were enriched (Figure 8B). The enrichment analysis results of other MRSB corresponding genes were presented in the Supplementary Material.

## Discussion

In this study, we identified genes with methylated CpGs which were associated with post-surgical treatment BLCA progression based on the analysis of BLCA TCGA DNA methylation data using two computational analysis algorithms, the SVM-RFE and iterative LASSO algorithms. Consequently, eight genes with specific CpG methylations were selected to build a MRSB in the training cohort, which was validated in the validation cohort. We demonstrated that MRSB was able to classify BLCA patients into high- or low-risk disease progression subgroups. Moreover, our data also showed that MRSB could predict the risks of disease recurrence and patient OS. Therefore, MRSB has great potential to be used to predict the post-surgery progression risk of BLCA, and it may provide novel insight into BLCA progression and the opportunity of stratified therapeutic strategy.

On the basis of MRSB, low-risk patients can avoid the toxic adverse effects of adjuvant treatment, while high-risk patients will be selected to receive active surveillance and intensified regimens to prevent tumor progression (Tsao et al., 2012). As shown in Figure 5A, the risk of disease progression in high-risk patients showed a bimodal distribution, and most disease progression was clustered within 9–10 months or 30 months after resection, which may reflect the true progression that had disseminated from the original cancer. There was a consistently low progression risk in low-risk patients, but at 20 months, it was relatively high compared to the other follow-up periods. Meanwhile, the death of patients from cancer was clustered within the 20th month during the follow-up, a potential consequence from the early disease progression from the high-risk disease progression group. Thus, MRSB may help to develop individualized disease progression monitoring and prevention strategies for BLCA patients. For patients with high risk of progression, they may be intensively monitored by screening of possible disease progression before 9–10 months and around 2.5 years after the treatment. The post-surgery disease monitoring follow-up may be safely reduced for patients with low progression risk, especially after the 20th month of resection, when their risk of disease progression and death will be significantly reduced.

Recently, three genome-wide studies have reported DNA methylation in BLCA. In the first study, four specific methylation regions were identified to predict the progression potential of NMIBC to MIBC by analyzing 192 patients with primary pTaG1/G2 BLCA. The area under the curve for GATA binding protein 2 (GATA2) was 0.803, for T-Box transcription factor 2 (TBX2) was 0.644, for T-Box transcription factor 3 (TBX3) was 0.785, and for Zic family member 4 (ZIC4) was 0.692, respectively (Beukers et al., 2015). This promising methylation biomarker developed from a limited number of samples yet was required to be validated in a much larger sample cohort. The second study developed an integrative framework between methylation and miRNAs associated with prognosis, but there was no appropriate validation cohort to verify the signature (Shivakumar et al., 2017). The last study, which showed that RSPH9 methylation was a potential prognostic predictor in NMIBC patients, used even less samples (NMIBC = 18, NC = 6) than the earlier studies and lacked validation analysis (Yoon et al., 2016).

Our study has the following advantages over the previous studies. We adopt a strategy to select reliable markers by combining the results of two different algorithms, which as much as possible minimized the loss or neglect of important markers compared with the method of using only a single strategy as in previous studies. The main statistical concern faced when methylation data are used to develop prognostic models is the processing of large quantities of markers yielded from ultrahigh-dimensional data. Overfitting of the overly vast and complex methylation signal model in the face of limited heterogeneity of the training cohort compromises the independent predictive efficacy of the model (Sveen et al., 2012). Parameters tuned during cross-validation in penalization of the methylation signal data can reduce this concern (van Houwelingen et al., 2006). LASSO can complete penalization and feature selection simultaneously (Tibshirani, 1996). The SVM-RFE algorithm may be more effective than linear discriminant analysis and mean squared error methods in identifying related features and in reducing redundant features (Xu et al., 2014). This combined analysis strategy not only reduces the number of potential false positive methylation features but also avoids redundancy in prognostic correlations between features.

More importantly, we established a nomogram with MRSB, age, sex, and tumor clinical stage to predict individual progression risk. The nomogram was reliable for predicting survival and recurrence risk. Therefore, our nomogram provides a potentially accurate prognostic indicator for patients with BLCA, which may be used to guide individualized post-surgery disease progression monitoring and prevention strategies.

Moreover, we found that high-level expression of the MRSB component-relevant genes TNFAIP8L3 and APC, which negatively correlated with their methylation status, was correlated with a poor prognosis. Generally, tumorigenesis is influenced by transcriptional activation of oncogenes *via* global DNA hypomethylation, while hypermethylation is commonly found at the promoters of tumor suppressors to silence their expression (Van Tongelen et al., 2017; Saghafinia et al., 2018). While TNFAIP8L3 has been reported with an oncogene role, APC is a well-established tumor suppressor gene. Therefore, the correlation of low methylation and high expression levels of both an oncogene (TNFAIP8L3) and a tumor suppressor gene (APC) with poor prognosis of BLCA is unexpected.

TNFAIP8L3 has been shown to promote the progression of gastric cancer, which can be suppressed by miR-9-5p (Fan et al., 2019). However, the role of TNFAIP8L3 and its methylation has not been reported in BLCA. According to our study, TNFAIP8L3 methylation level provides a negative contribution to MRSB model, which is consistent to the overexpression of TNFAIP8L3 at both the mRNA and protein levels in poor-prognosis BLCA cases. Therefore, overexpression of TNFAIP8L3, caused by hypomethylation, may contribute to BLCA progression or resistance to therapies. MAPK signaling and JAK-STAT signaling pathways have been shown to have vital roles in BLCA progression and might be connected with TNFAIP8L3 activation (Huang et al., 2020; Lei et al., 2020), but the mechanisms connecting TNFAIP8L3 and these two pathways remain unclear. Further studies are required.

In colorectal cancer, APC is a well-established tumor suppressor, and its inactivation is a common mechanism of colorectal tumorigenesis. APC mutation has been associated with the activation of Wnt/β-catenin signaling pathway (Qu et al., 2018). In breast cancer, APC mutation has been associated with overexpression and reactivation of the poor prognosis and tumor metastasis-associated ErbB receptor (Wang, 2017). However, in our study of BLCA, although we revealed that the methylation and expression change of APC influenced BLCA progression through Wnt/β-catenin signaling and ErbB receptor pathways, suppression of APC expression by DNA methylation was associated with good disease progression so that cases with poor prognosis had relatively high expression and low methylation level of APC. Our observation, which may conflict with the role of APC in colorectal and breast cancers, is supported by previous DNA methylation studies of BLCA for clinical prognosis. Reduced methylation of the APC promoter region has been previously reported as an independent poor prognostic biomarker of BLCA (Eissa et al., 2011), and in a recent study, reduced APC methylation was associated with the progression of BLCA, although not with OS (Bai et al., 2019). The potential explanation may be that the reduced function of APC by methylation may contribute to the tumorigenesis but not progression of BLCA, and BLCA cases that developed without inactivating the APC pathway may have accumulated other genetic changes associated with poor disease prognosis. This is an interesting observation in BLCA, and further mechanistic investigations are warranted.

Our study has the following limitations. First, our study collected patients with different disease stages. Whether MRSB is affected by heterogeneity among patients with early or advanced stage BLCA requires further investigation. Second, the biological mechanisms of the involvement of certain methylated genes, such as KRTDAP, ZC3H3, and PI3, are yet to be investigated. Third, because the TCGA patients were mainly from the United States and most of the samples were mainly from Caucasians, independent external validation on more diverse patient populations is necessary for the global application of MRSB developed in this study. Our results warrant further investigation in a larger independent cohort.

In conclusion, MRSB, an eight-genes-based DNA methylation signature, is an efficient prognostic biomarker to predict the progression risk of BLCA patients. The nomogram including MRSB may provide individualized BLCA patient monitoring and prevention strategies. Our study not only indicates the potential value of MRSB as a prognostic predictor in BLCA but also points to a novel direction for further mechanistic research of BLCA progression.

## Data Availability Statement

Publicly available datasets were analyzed in this study. The raw data of DNA methylation and gene expression data in this study are available in TCGA-BLCA program (https://portal.gdc.cancer.gov).

## Ethics Statement

The studies involving human participants were reviewed and approved by the Ethics Committee of Zhengzhou University. The patients/participants provided their written informed consent to participate in this study.

## Author Contributions

DS, Y-JL, LZ, and YFG performed the conception and design. YDG, PC, YC, and CH performed the data collection and processing. YFG and Y-JL performed the data analysis and interpretation. JY, YD, and YFG performed the experiments. YFG, JY, Y-JL, LZ, and DS performed the manuscript writing. All authors discussed the results and reviewed the manuscript.

## Conflict of Interest

The authors declare that the research was conducted in the absence of any commercial or financial relationships that could be construed as a potential conflict of interest.

## Publisher’s Note

All claims expressed in this article are solely those of the authors and do not necessarily represent those of their affiliated organizations, or those of the publisher, the editors and the reviewers. Any product that may be evaluated in this article, or claim that may be made by its manufacturer, is not guaranteed or endorsed by the publisher.

## Footnotes

1
https://www.cancer.gov/tcga

2
http://www.gsea-msigdb.org/gsea/msigdb

## Supplementary Material

Supplementary Figure 1 Net decision curve analyses demonstrating the benefit for the MRSB and the optimal clinical covariates for prognosis. **(A)** For bladder cancer patients, the turquoise line is the net benefit of providing all patients with the prediction of the nomogram, and the horizontal black line is the net benefit of providing no patients with the prediction of the nomogram. The net benefits provided by each nomogram with different clinical covariates are given (MRSB: methylation risk score for bladder cancer, stage: pathologic tumor stage, grade: histological grade). Model 1=Risk+age+gender+stage, model 2=Risk+age+gender+grade, model 3=Risk+age+stage, model 4=Risk+age+grade, model 5=Risk+gender+stage, model 6=Risk+gender+grade, model 7=Risk+stage, and model 8=Risk+grade. **(B)** the net reduction analyses demonstrate how many patients with the prediction of the nomogram could avoid interventions without missing disease progression events within 1 year.

Supplementary Figure 2 The nomograms for overall survival based on the MRSB.

Supplementary Figure 3 The nomograms for recurrence-free survival based on the MRSB.

Supplementary Figure 4 The expression of the MRSB component-related genes was associated with the prognosis in BLCA patients. **(A)** Performance of the MRSB in PFS. **(B)** Performance of the MRSB in OS.

Supplementary Table 1 One hundred thirty CpGs were identified by the SVM-RFE algorithm.

Supplementary Table 2 Fifteen CpGs that appeared more than 500 times in 1,000 iterations and were considered consensus CpGs that distinguished high- from low-risk groups were identified by the iterative LASSO algorithm.

Supplementary Table 3 Gene set enrichment analysis (GSEA) results of MRSB corresponding genes in TCGA BLCA cohort.
