# Supplementary figures and images for: A Novel CpG Methylation Risk Indicator for Predicting Prognosis in Bladder Cancer
Source: Front Cell Dev Biol. 2021 Sep 1;9:642650. doi: 10.3389/fcell.2021.642650 (PMC8440888; doi:10.3389/fcell.2021.642650)

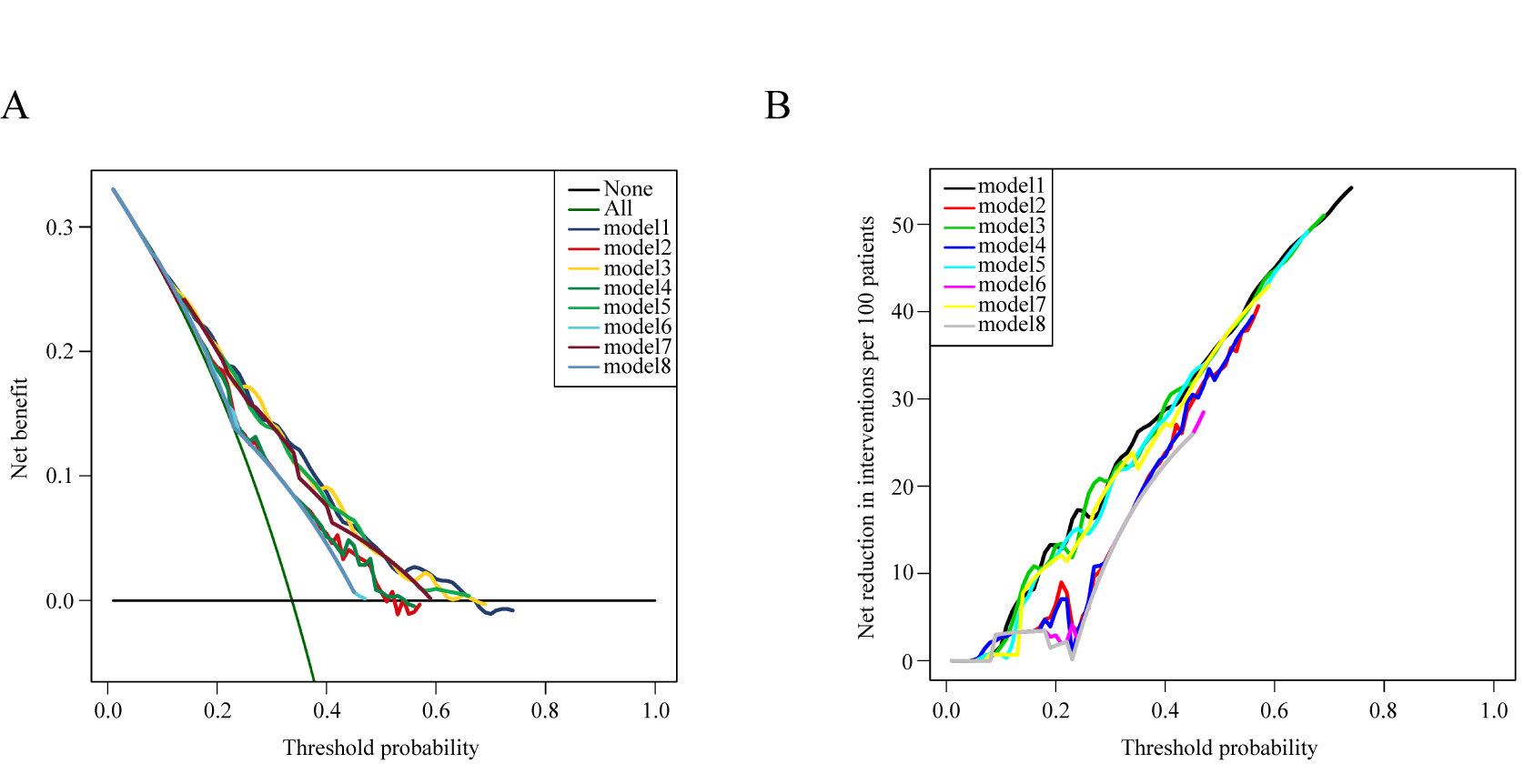

Supplement: Supplementary Figure 1 — Net decision curve analyses demonstrating the benefit for the MRSB and the optimal clinical covariates for prognosis. (A) For bladder cancer patients, the turquoise line is the net benefit of providing all patients with the prediction of the nomogram, and the horizontal black line is the net benefit of providing no patients with the prediction of the nomogram. The net benefits provided by each nomogram with different clinical covariates are given (MRSB: methylation risk score for bladder cancer, stage: pathologic tumor stage, grade: histological grade). Model 1=Risk+age+gender+stage, model 2=Risk+age+gender+grade, model 3=Risk+age+stage, model 4=Risk+age+grade, model 5=Risk+gender+stage, model 6=Risk+gender+grade, model 7=Risk+stage, and model 8=Risk+grade. (B) the net reduction analyses demonstrate how many patients with the prediction of the nomogram could avoid interventions without missing disease progression events within 1 year. [file Image_1.TIF]

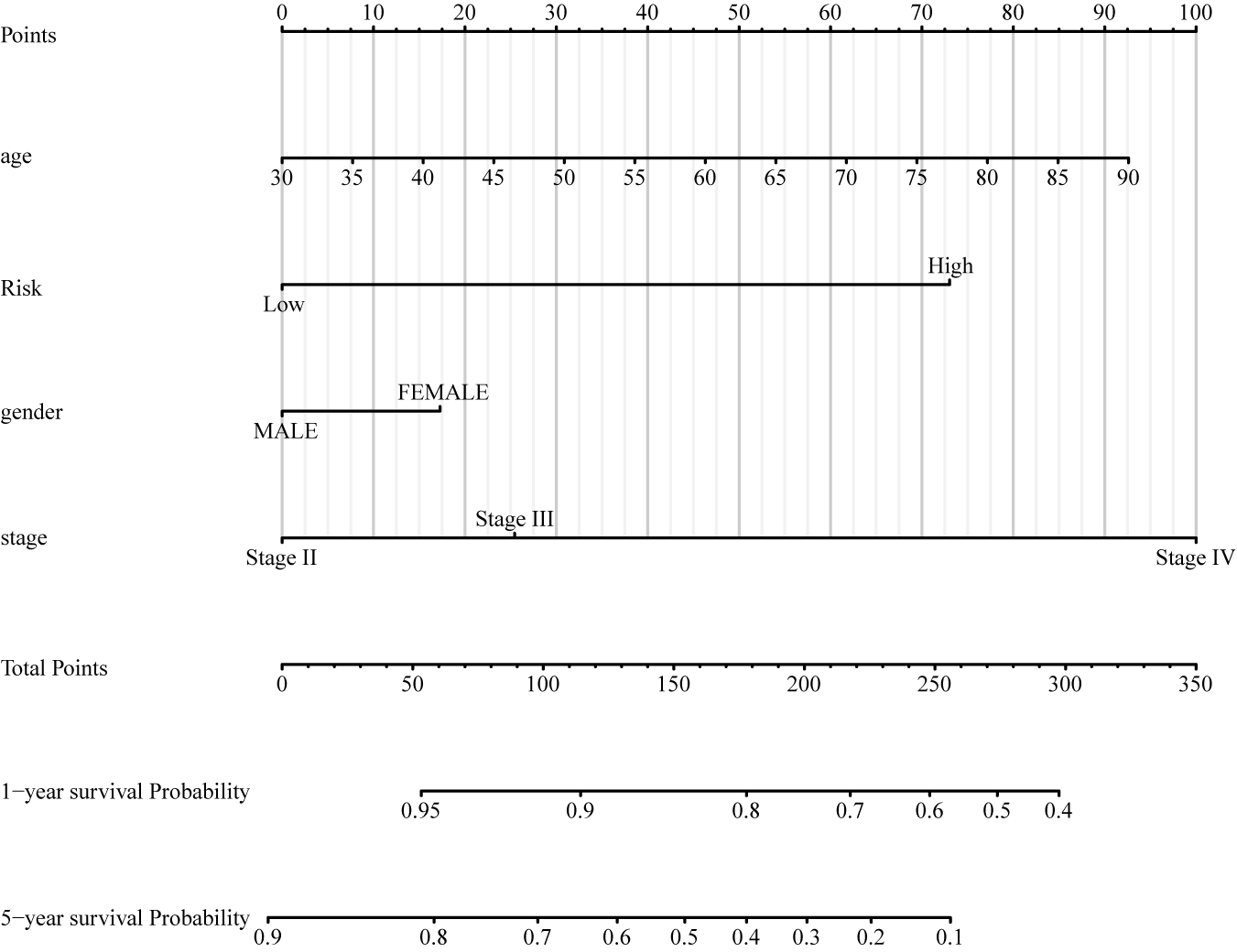

Supplement: Supplementary Figure 2 — The nomograms for overall survival based on the MRSB. [file Image_2.TIF]

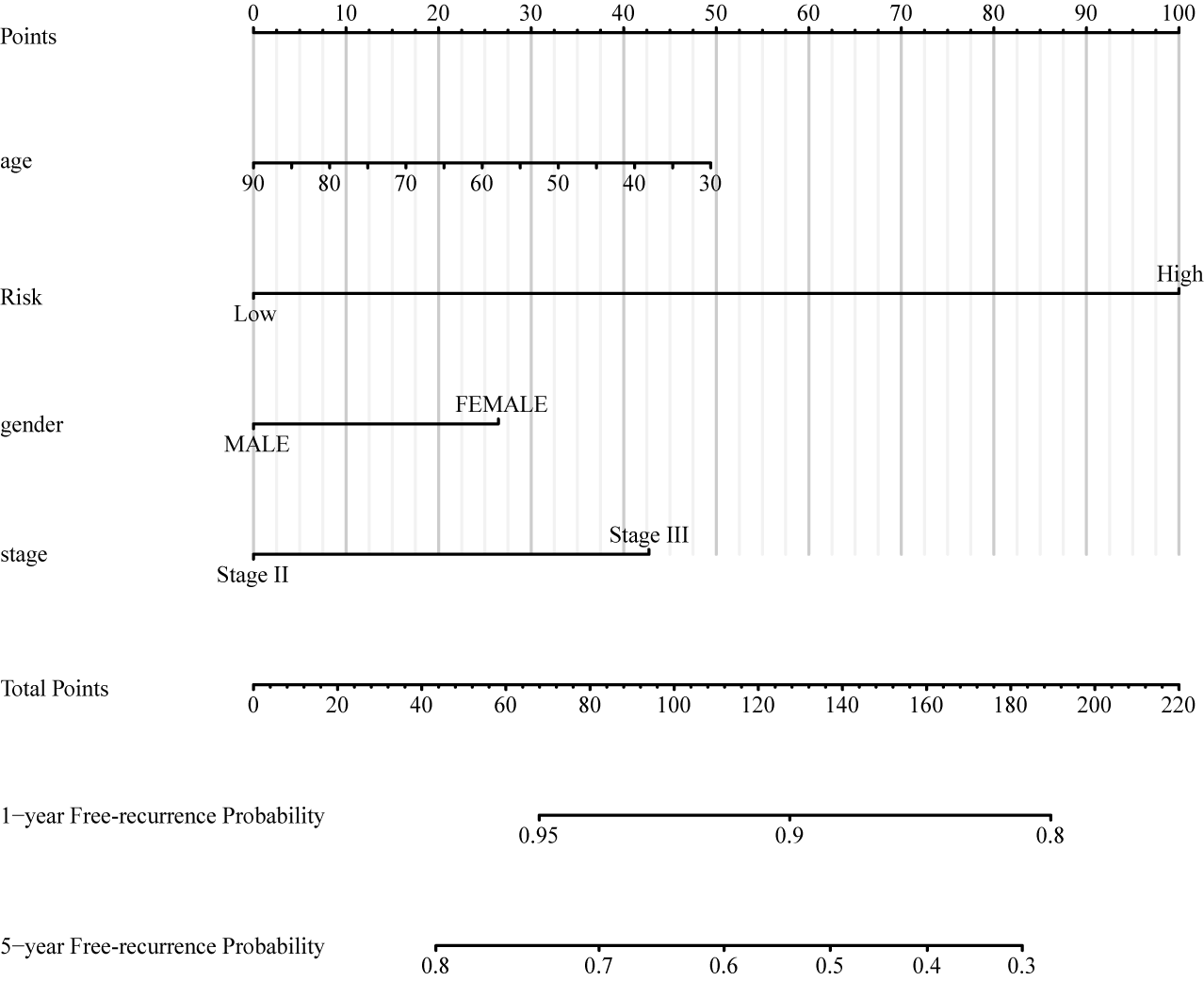

Supplement: Supplementary Figure 3 — The nomograms for recurrence-free survival based on the MRSB. [file Image_3.TIF]

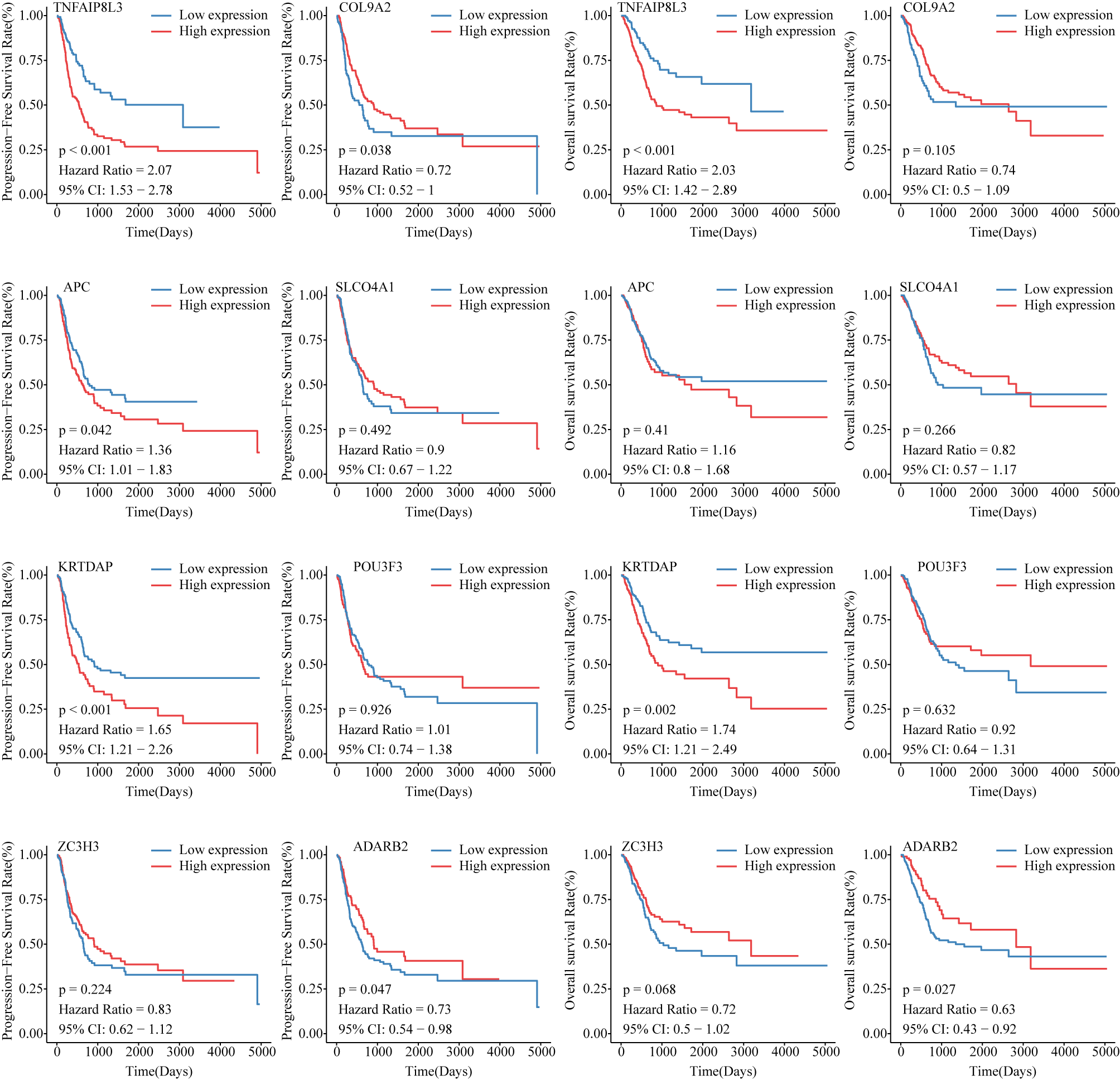

Supplement: Supplementary Figure 4 — The expression of the MRSB component-related genes was associated with the prognosis in BLCA patients. (A) Performance of the MRSB in PFS. (B) Performance of the MRSB in OS. [file Image_4.TIF]
